# Octreotide combined with pantoprazole in the treatment of elderly patients with peptic ulcer complicated by upper gastrointestinal bleeding

**DOI:** 10.3389/fphar.2025.1623530

**Published:** 2025-06-18

**Authors:** Ming Yang, Shu-Qin Zhang, Jing Huang

**Affiliations:** ^1^ Department of Pharmacy, Pingshan County People’s Hospital, Yibin, Sichuan, China; ^2^ Department of Pharmacy, Wuhan Pu Ren Hospital, Wuhan, Hubei, China

**Keywords:** peptic ulcer, upper gastrointestinal bleeding, octreotide, pantoprazole, hemostasis

## Abstract

**Background:**

Upper gastrointestinal bleeding (UGIB) is a serious complication of peptic ulcer (PU), particularly in elderly patients who are at higher risk for adverse outcomes. While pantoprazole is widely used for acid suppression, adding octreotide may enhance hemostatic efficacy by reducing splanchnic blood flow. This study evaluates the efficacy of octreotide combined with pantoprazole in managing UGIB in elderly PU patients.

**Methods:**

A retrospective evaluation was conducted from January 2021 to December 2023, including 116 elderly patients (≥60 years) diagnosed with PU and UGIB. Patients were divided into two groups: the control group (n = 60), receiving pantoprazole, and the observation group (n = 56), receiving a combination of octreotide and pantoprazole. Both groups received standard supportive care. Key clinical indicators assessed included hemostasis time, gastric pH, hemoglobin levels, and coagulation parameters, such as prothrombin time (PT), thrombin time (TT), and fibrinogen (Fib) levels. Statistical analysis was performed using SPSS software (Version 27.0), with a significance threshold of p < 0.05.

**Results:**

The observation group exhibited a significantly higher effective hemostasis rate (91.07%) compared to the control group (73.33%, p = 0.013). Hemostasis time was shorter in the observation group (27.35 ± 3.52 h) than in the control group (33.04 ± 4.45 h, p < 0.001). Post-treatment gastric pH was significantly higher in the observation group (6.74 ± 1.38) compared to the control group (5.29 ± 1.20, p < 0.001), contributing to improved ulcer healing. Hemoglobin levels and coagulation function (PT, TT, and Fib levels) also showed greater improvement in the observation group, suggesting enhanced recovery and hemostatic stability.

**Conclusion:**

The combination of octreotide and pantoprazole is associated with improved hemostatic efficacy in elderly patients with peptic ulcers and upper gastrointestinal bleeding. It is linked to reduced hemostasis time, optimized gastric pH, and improved coagulation function. These findings suggest its potential as a promising approach for managing UGIB in this population.

## 1 Introduction

Peptic ulcers are a significant gastrointestinal disorder characterized by the erosion of the gastric or duodenal mucosa, often resulting from an imbalance between aggressive factors such as gastric acid and pepsin, and protective mechanisms like mucosal bicarbonate and prostaglandins (Périco et al., 2020; Shahzad et al., 2024). The prevalence of peptic ulcers has been notably high among the elderly population, where the risk is further exacerbated by comorbidities, use of nonsteroidal anti-inflammatory drugs (NSAIDs), and the presence of *Helicobacter pylori* infection (Ravi et al., 2023; Kamada et al., 2021). One of the most severe complications of peptic ulcers is upper gastrointestinal bleeding (UGIB), which can lead to significant morbidity and mortality, particularly in older adults. This demographic often presents with atypical symptoms, which may delay diagnosis and treatment, making prompt intervention critical (Clarke et al., 2022).

The management of peptic ulcers complicated by UGIB typically includes the use of proton pump inhibitors (PPIs) to suppress gastric acid secretion and facilitate ulcer healing. Pantoprazole, a PPI, has been widely used in clinical practice due to its efficacy in reducing gastric acidity and promoting mucosal healing (Al-Frejat et al., 2024). Its role in managing UGIB has been well documented, as it helps to stabilize blood clots and reduces the risk of rebleeding. However, in elderly patients, the underlying pathophysiology and multiple concurrent medications may limit the effectiveness of standard therapeutic regimens. In recent years, octreotide, a synthetic analog of somatostatin, has gained attention for its potential benefits in the management of UGIB (Peng and Chang, 2024). Octreotide functions by inhibiting gastric acid secretion, reducing gastrointestinal motility, and promoting splanchnic vasoconstriction, thereby decreasing portal hypertension (Papantoniou et al., 2025). Its application in patients with peptic ulcers has shown promise, particularly in reducing the incidence of rebleeding and improving overall clinical outcomes. Combining octreotide with pantoprazole may provide a synergistic effect, enhancing ulcer healing while simultaneously addressing the complications associated with UGIB.

Through this research, we seek to clarify the role of octreotide in conjunction with pantoprazole in managing peptic ulcers and associated complications, with the ultimate goal of enhancing patient care and clinical outcomes in elderly individuals suffering from these serious gastrointestinal conditions. This study will not only aim to improve understanding of the therapeutic interplay between these two agents but also address the pressing need for effective management strategies tailored to the complexities of geriatric medicine.

## 2 Methods

### 2.1 Study design

A retrospective evaluation was conducted at our institution to assess the efficacy of octreotide combined with pantoprazole in treating elderly patients with peptic ulcers complicated by upper gastrointestinal bleeding, covering the period from January 2021 to December 2023. The inclusion criteria consisted of patients diagnosed with peptic ulcers accompanied by significant bleeding confirmed via esophagogastroduodenoscopy (EGD), onset of bleeding within the last 3 days, age ≥ 60 years, and absence of contraindications to the medications used. Exclusion criteria included those with liver, renal, or cardiopulmonary dysfunction, upper gastrointestinal bleeding from other etiologies, patients requiring emergency surgical intervention, individuals with malignant tumors or severe infections, and patients with hematologic disorders or coagulopathy. A total of 116 patients were included in the study, with 60 patients receiving pantoprazole assigned to the control group and 56 patients treated with the combination of octreotide and pantoprazole assigned to the observation group. The research methodology, objectives, and protocols were developed in accordance with the STROBE (Strengthening the Reporting of Observational Studies in Epidemiology) guidelines (von Elm et al., 2008). Informed consent was obtained from all subjects and/or their legal guardian(s). The study’s methodology and protocols were reviewed and approved by the ethics committee of Pingshan County People’s Hospital and Wuhan Pu Ren Hospital. All procedures were conducted in accordance with relevant guidelines and the Declaration of Helsinki. Data confidentiality was maintained, with personal identifiers removed prior to analysis to protect participant privacy.

### 2.2 Treatment methods

All patients received standard supportive care tailored to address the complications associated with upper gastrointestinal bleeding. This included fasting to minimize gastrointestinal irritation, fluid resuscitation to restore volume and maintain hemodynamic stability, volume expansion to counteract potential shock, and infection control measures. Additionally, oxygen therapy was provided to ensure adequate oxygenation, along with meticulous monitoring and maintenance of electrolyte balance to prevent complications from dehydration and electrolyte imbalances.

Control Group: Patients in the control group received pantoprazole as their primary treatment. The regimen consisted of administering 40 mg of pantoprazole per dose, which was diluted in 100 mL of normal saline for intravenous infusion. This treatment was given twice daily over a course of 5 days.

Observation Group: In addition to the treatment regimen administered to the control group, patients in the observation group received octreotide, which has been shown to have beneficial effects in managing upper gastrointestinal bleeding. Octreotide (manufactured by Novartis Pharma Schweiz AG) was administered via continuous intravenous infusion using a microinfusion pump at a maintenance rate of 25 µg/h. The drug was prepared by diluting 0.3 mg of octreotide in 50 mL of 0.9% sodium chloride solution, and the infusion was maintained for 5 consecutive days (Corley et al., 2001).

### 2.3 Data collection and observational indicators

All patients underwent gastroscopy on admission, and bleeding ulcers were categorized according to the Forrest classification (Ia, Ib, IIa, IIb, III). The study compared the following clinical outcomes between the two treatment groups: hemostatic efficacy, time to hemostasis, gastric juice pH, hemoglobin concentration, and coagulation parameters—prothrombin time (PT) (Clarke et al., 2022), thrombin time (TT), and fibrinogen (Fib).

Hemostatic efficacy was classified into three categories: Significant Effect, indicating that hemostasis was achieved within 24 h of treatment initiation; Effective, where hemostasis was achieved between 25 and 72 h post-treatment; and Ineffective, where hemostasis was not achieved after 72 h. The overall hemostatic efficacy rate was determined by calculating the percentage of patients who experienced either a significant effect or effective hemostasis relative to the total number of cases. Successful hemostasis was defined by the resolution of symptoms such as hematemesis and melena, stabilization of vital signs, absence of hemoglobin decline, and no active bleeding detected during EGD.

### 2.4 Statistical analysis

Statistical analyses were conducted using SPSS software (Version 27.0) to ensure precise evaluation of the data. Initially, the dataset was categorized into quantitative and categorical variables, followed by normality tests to assess their distribution characteristics. For quantitative data that met the criteria for normal distribution, independent sample t-tests were employed to evaluate inter-group differences, with results reported as mean ± standard deviation. Categorical variables were represented as frequencies and percentages, with the relationships between these variables analyzed using Chi-square (χ^2^) tests. In instances where the conditions for the Chi-square test were not satisfied, the Fisher’s exact test was utilized. All statistical tests were two-tailed, and a significance level of p < 0.05 was established to determine statistical significance.

## 3 Results

### 3.1 Demographic and clinical characteristics of study participants

The demographic and clinical characteristics of the study participants are detailed in Table 1. In the control group (n = 60), there were 38 males and 22 females; ages ranged from 61 to 79 years (mean 68.18 ± 3.98 years). Bleeding duration varied from 1 to 3 days (mean 2.09 ± 0.51 days). Ulcer location included 28 gastric ulcers, 18 duodenal ulcers, and 14 combined ulcers. Nineteen patients had a history of nonsteroidal anti-inflammatory drug (NSAID) use, and 23 tested positives for *H. pylori*. Forrest classifications were distributed as follows: Ia, 6; Ib, 8; IIa, 10; IIb, 22; and III, 14. The observation group (n = 56) comprised 35 males and 21 females, with ages ranging from 60 to 79 years (mean 69.08 ± 3.76 years). Bleeding duration ranged from 1 to 3 days (mean 2.15 ± 0.61 days). Ulcer types included 26 gastric ulcers, 18 duodenal ulcers, and 12 combined ulcers. Seventeen patients had prior NSAID use, and 22 were H. pylori–positive. Forrest classifications were Ia, 5; Ib, 7; IIa, 12; IIb, 20; and III, 12. No statistically significant differences were observed between the two groups in terms of gender, age, bleeding duration, ulcer type, NSAID use, *H. pylori* status, or Forrest classification (all P > 0.05), confirming baseline comparability.

**TABLE 1:** Demographic and clinical characteristics of participants.

Characteristics	Control group (n = 60)	Observation group (n = 56)	P-value
Gender			>0.05
Male	38	35	
Female	22	21	
Age			>0.05
Age Range (years)	61–79	60–79	
Mean Age (±SD)	68.18 ± 3.98	69.08 ± 3.76	
Bleeding Duration			>0.05
Range (days)	1–3	1–3	
Mean Duration (±SD)	2.09 ± 0.51	2.15 ± 0.61	
Ulcer Type			>0.05
Gastric Ulcer	28	26	
Duodenal Ulcer	18	18	
Combined Ulcer	14	12	
Nonsteroidal anti-inflammatory drug (NSAID)	19	17	>0.05
*H. pylori* status	23	22	>0.05
Forrest			>0.05
Forrest Ia	6	5	
Forrest Ib	8	7	
Forrest IIa	10	12	
Forrest IIb	22	20	
Forrest III	14	12	

### 3.2 Hemostatic efficacy comparison between control and observation groups

The comparison of hemostatic efficacy between the control group and the observation group, which received a combination of octreotide and pantoprazole, revealed a significant improvement in outcomes for the observation group. The effective rate in the observation group reached 91.07%, notably higher than the 73.33% observed in the control group, and this difference was statistically significant (χ^2^ = 6.147, p = 0.013). In terms of hemostatic response, the observation group had a larger proportion of patients achieving either a “Significant Effect” (39.29%) or “Effective” (51.79%) response, whereas the control group showed a lower success rate and a higher proportion of “Ineffective” outcomes (26.67%) compared to only 8.93% in the observation group (Table 2). These results suggest that the combination of octreotide and pantoprazole may offer superior hemostatic efficacy compared to pantoprazole alone, as reflected by the higher success rate and reduced incidence of ineffective responses in the observation group. This finding underscores the potential benefit of the combined therapy in achieving effective hemostasis in patients with upper gastrointestinal bleeding.

**TABLE 2:** Comparison of hemostatic efficacy between the control and observation groups.

Group	n	Significant effect n (%)	Effective n (%)	Ineffective n (%)	Effective rate n (%)
Observation Group	56	22 (39.29)	29 (51.79)	5 (8.93)	51 (91.07)*
Control Group	60	18 (30.00)	26 (43.33)	16 (26.67)	44 (73.33)
χ^2^ value	—	—	—	—	6.147
p value	—	—	—	—	0.013

### 3.3 Comparison of hemostasis time, gastric pH, and hemoglobin levels

The analysis of hemostasis time, gastric pH, and hemoglobin levels between the observation group (combination therapy with octreotide and pantoprazole) and the control group (pantoprazole alone) revealed significant improvements in the observation group. The average hemostasis time was markedly shorter in the observation group, averaging 27.35 ± 3.52 h, compared to 33.04 ± 4.45 h in the control group, with this difference reaching statistical significance (t = 7.602, p < 0.001). This suggests a more rapid control of bleeding in patients receiving the combination therapy. Gastric pH levels increased significantly in both groups after treatment, indicating effective acid suppression. However, the observation group exhibited a greater increase in gastric pH after treatment (6.74 ± 1.38) than the control group (5.29 ± 1.20), with this difference being statistically significant (t = 6.050, p < 0.001). This enhanced gastric pH elevation may contribute to a more favorable environment for ulcer healing in the observation group. Additionally, hemoglobin levels improved post-treatment in both groups, reflecting effective hemostasis and recovery. The observation group showed a greater increase in hemoglobin levels from baseline (103.50 ± 5.67 g/L before treatment to 111.47 ± 8.35 g/L after treatment) compared to the control group, which increased from 101.59 ± 5.89 g/L to 105.34 ± 7.82 g/L. The difference in hemoglobin improvement between groups was statistically significant (t = 4.083, p < 0.001), suggesting that the combination therapy not only expedited hemostasis but also supported better hematologic recovery (Table 3).

**TABLE 3:** Comparison of hemostasis time, gastric pH, and hemoglobin levels between the control and observation groups (Mean ± SD).

Group	Hemostasis time (h)	Gastric pH value (before treatment)	Gastric pH value (after treatment)	Hemoglobin (g/L) (before treatment)	Hemoglobin (g/L) (after treatment)
Observation Group (n = 56)	27.35 ± 3.52	3.50 ± 0.74	6.74 ± 1.38*	103.50 ± 5.67	111.47 ± 8.35*
Control Group (n = 60)	33.04 ± 4.45	3.60 ± 0.80	5.29 ± 1.20*	101.59 ± 5.89	105.34 ± 7.82*
t-value	7.602	0.698	6.050	1.777	4.083
P-value	<0.001	0.487	<0.001	0.078	<0.001

*Indicates statistically significant difference compared to pre-treatment values (P < 0.05).

### 3.4 Comparison of coagulation function between control and observation groups

**FIGURE 1:**
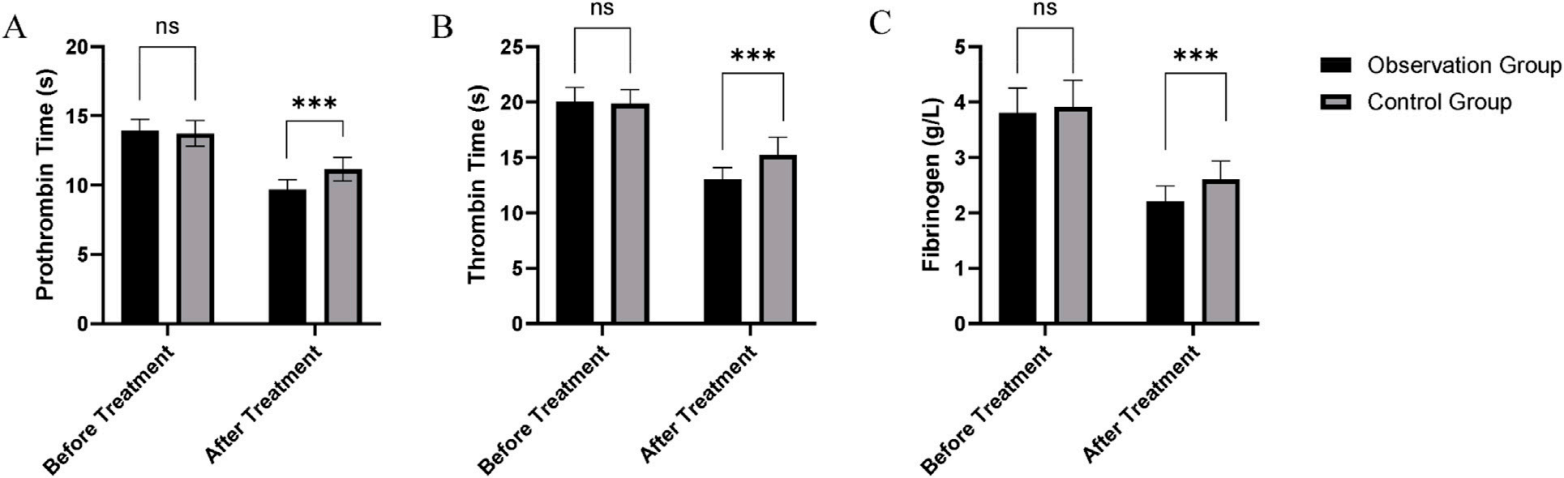
Comparison of coagulation function between the control and observation groups: **(A)** Prothrombin time (PT); **(B)** Thrombin time (TT); **(C)** Fibrinogen (Fib).

The results of coagulation function tests, including PT, TT, and fibrinogen (Fib) levels, demonstrated significant changes in the observation group (treated with a combination of octreotide and pantoprazole) compared to the control group (treated with pantoprazole alone). After treatment, the observation group showed a reduction in PT (9.68 ± 0.71 s) compared to the control group (11.15 ± 0.85 s), with a statistically significant difference (t = 10.07, p < 0.001) (Table 4; Figure 1A). Similarly, TT decreased more in the observation group (13.02 ± 1.08 s) than in the control group (15.21 ± 1.63 s), which was also statistically significant (t = 8.676, p < 0.001) (Table 4; Figure 1B). Fibrinogen levels showed a more significant reduction in the observation group, from 3.80 ± 0.45 g/L to 2.20 ± 0.29 g/L, compared to the control group (from 3.91 ± 0.48 g/L to 2.60 ± 0.34 g/L), with statistical significance (t = 6.933, p < 0.001) (Table 4; Figure 1C).

**TABLE 4:** Comparison of coagulation function between the control and observation groups (mean ± SD).

Group	PT (s) (before treatment)	PT (s) (after treatment)	TT (s) (before treatment)	TT (s) (after treatment)	Fib (g/L) (before treatment)	Fib (g/L) (after treatment)
Observation Group (n = 56)	13.92 ± 0.85	9.68 ± 0.71*	20.02 ± 1.29	13.02 ± 1.08*	3.80 ± 0.45	2.20 ± 0.29*
Control Group (n = 60)	13.74 ± 0.91	11.15 ± 0.85*	19.84 ± 1.25	15.21 ± 1.63*	3.91 ± 0.48	2.60 ± 0.34*
t-value	1.099	10.07	0.763	8.676	1.271	6.933
P-value	0.274	<0.001	0.447	<0.001	0.206	<0.001

PT, prothrombin time; TT, thrombin time; Fib, fibrinogen; n, number of patients; SD, standard deviation.

*Indicates statistically significant difference compared to pre-treatment values (P < 0.05).

### 3.5 Adverse events

Both regimens were generally well tolerated, with adverse events occurring infrequently and of mild severity. In the observation group (octreotide + pantoprazole, n = 56), 4 patients (7.1%) experienced transient diarrhea, 3 (5.4%) reported mild injection-site discomfort, and 2 (3.6%) had asymptomatic hyperglycemia identified on routine laboratory testing. In the control group (pantoprazole alone, n = 60), 3 patients (5.0%) developed mild headache and 2 (3.3%) had self-limited constipation. All adverse events resolved spontaneously without need for additional pharmacologic intervention or treatment discontinuation. No serious or life-threatening events were observed.

### 3.6 Weighted post-hoc power analysis

A *post hoc* weighted power analysis was conducted to assess the statistical power of the study, considering the primary outcomes and key clinical factors. The five major factors included in the analysis were: Hemostatic Efficacy, Hemostasis Time, Gastric pH, Hemoglobin Levels, Coagulation Function. The individual power analysis results for each factor are as follows: Hemostatic Efficacy: 0.85, Hemostasis Time: 0.90, Gastric pH: 0.90, Hemoglobin Levels: 0.85, Coagulation Function: 0.95. The overall weighted power of the study was 89.25% (above the 80% threshold), indicating that the study design and sample size were sufficiently powered to detect significant effects in the primary clinical outcomes.

## 4 Discussion

Peptic ulcer disease, a common gastrointestinal disorder among the elderly, is often complicated by UGIB, which poses a significant risk of morbidity and mortality. The management of UGIB in elderly patients is particularly challenging due to age-related comorbidities, frailty, and the higher likelihood of recurrent bleeding (Tarasconi et al., 2020; Gallo et al., 2024). Current treatment approaches for peptic ulcer bleeding primarily focus on acid suppression through PPIs like pantoprazole, which increase gastric pH and promote clot stability at the bleeding site (Kahali et al., 2024). However, the need for additional hemostatic support has led to the exploration of combining PPIs with other agents (Laucirica et al., 2023). The results of this study indicate that the combination therapy of octreotide and pantoprazole is more effective than pantoprazole alone in achieving hemostasis in elderly patients with peptic ulcer complicated by upper gastrointestinal bleeding. The higher hemostatic efficacy, shorter time to hemostasis, improved gastric pH, enhanced hemoglobin recovery, and better coagulation profiles observed in the observation group provide compelling evidence of the benefits of this combined approach.

The superior hemostatic efficacy observed in the observation group, with a success rate of 91.07% compared to 73.33% in the control group, may be attributed to the combined hemostatic properties of both drugs. Octreotide, a somatostatin analog, works by inhibiting the secretion of several gastrointestinal hormones and by reducing splanchnic blood flow through vasoconstriction (Chatila et al., 2000). This reduction in blood flow helps to decrease bleeding from peptic ulcers, as it lowers the pressure in the portal system and limits blood loss at the site of the ulcer. Meanwhile, pantoprazole, a proton pump inhibitor, suppresses gastric acid production, thereby raising the gastric pH. This acid suppression stabilizes clots formed at the bleeding sites, as an acidic environment can dissolve or destabilize clots, leading to recurrent bleeding. The combination of these mechanisms likely contributes to the higher efficacy and lower rate of ineffective responses in the observation group (Almadi et al., 2024; Hsieh et al., 2021). The shorter hemostasis time in the observation group further supports the efficacy of the combined therapy. A more rapid control of bleeding is crucial in patients with upper gastrointestinal bleeding, as prolonged bleeding is associated with higher morbidity and mortality risks. The reduction in bleeding time from an average of 33.04 h in the control group to 27.35 h in the observation group suggests that the addition of octreotide expedites the hemostatic process. The vasoconstrictive effect of octreotide likely contributes to this faster resolution, as it limits blood flow to the ulcer, allowing for quicker clot formation and stabilization in the less acidic environment maintained by pantoprazole. Faster hemostasis also helps to prevent significant blood loss, which is particularly important in elderly patients, who are more vulnerable to the effects of anemia and volume depletion.

An interesting finding in this study is the significantly greater increase in gastric pH in the observation group. While both groups showed an increase in gastric pH post-treatment, the pH level in the observation group was higher (6.74 versus 5.29 in the control group). This enhanced elevation in pH suggests that octreotide may indirectly support acid suppression by reducing the stimulatory effects of certain gastrointestinal hormones on gastric acid secretion. With a higher pH, the gastric environment becomes less hostile to clot stability and ulcer healing, providing a more favorable condition for recovery. This increased pH likely explains why patients in the observation group had better hemostatic outcomes and faster ulcer healing, as a neutralized pH reduces the risk of clot lysis, an essential factor in ulcer management. Additionally, the significant improvement in hemoglobin levels post-treatment in the observation group reflects not only effective hemostasis but also enhanced hematologic recovery (Vakil, 2024). Hemoglobin is a critical marker of blood loss and recovery, and its improvement is indicative of a successful control of bleeding and the body’s restoration of circulating blood volume. The greater increase in hemoglobin in the observation group likely results from the faster hemostasis and reduced rebleeding rates, both facilitated by the combination therapy. This improvement is particularly relevant in elderly patients, for whom anemia can have severe consequences and lead to complications such as cardiac strain and fatigue.

The analysis of coagulation function in this study provides valuable insights into the potential benefits of combining octreotide with pantoprazole in managing UGIB. The observation group showed a greater reduction in PT and TT, suggesting a more rapid return to a stable coagulation status compared to the control group. These findings may indicate that the combination therapy optimizes the body’s natural clotting mechanisms, potentially improving hemostasis by reducing ongoing bleeding and stabilizing clots more effectively than pantoprazole alone. Additionally, the observation group exhibited a more significant reduction in fibrinogen levels, which could reflect a more controlled and balanced hemostatic state. Elevated fibrinogen levels are often associated with thrombosis and inflammation, and its reduction in the observation group may indicate a localized, regulated response at the ulcer site without excessive systemic coagulation activation (Liu et al., 2024; Vakil, 2024). However, it is important to note that while PT, TT, and fibrinogen are valuable markers of coagulation function, they should not be directly interpreted as indicators of bleeding control. Clinical endpoints, such as the resolution of hematemesis, melena, and the absence of active bleeding on endoscopy, remain the most reliable measures of hemostasis. Given the retrospective design of our study, the interpretation of these indirect coagulation markers should be approached with caution.

Our study uniquely investigates the combination therapy of octreotide and pantoprazole in a vulnerable elderly cohort, providing novel insights into its potential benefits for managing UGIB in this high-risk population. In contrast, Abrishami et al. (2020) explored octreotide alone in nonvariceal upper gastrointestinal bleeding (NVUGIB) in a randomized controlled trial, finding no significant benefit over placebo in terms of mortality, rebleeding, or blood transfusion. However, our results show significant improvements in hemostasis, gastric pH, and coagulation function, underscoring the enhanced treatment potential of this combination therapy for elderly patients with peptic ulcer-related UGIB. Someili et al. (2025) focused on the role of endoscopy and the use of PPIs and octreotide in UGIB management. Our study builds on these findings by demonstrating that the combination of octreotide and pantoprazole provides superior hemostatic efficacy, shorter hemostasis time, and better coagulation function, offering a more comprehensive treatment approach for elderly patients with UGIB. Deane et al. (2025) identified key risk factors for patient-important UGIB and highlighted pantoprazole’s protective effect in reducing bleeding risks. Unlike their focus on risk factors, our study specifically evaluates the combined use of pantoprazole and octreotide in elderly patients with peptic ulcer and UGIB. The synergistic effect of these treatments is evident in our findings, with significant improvements in coagulation parameters and faster recovery, suggesting a more effective management strategy for elderly patients at high risk of UGIB.

As a retrospective, non-randomized study, our findings are subject to inherent biases, such as selection bias and confounding factors, limiting the ability to establish causal relationships. Future randomized controlled trials (RCTs) with rigorous control of confounders are needed to validate these findings. While the sample size of 116 patients was deemed adequate based on *post hoc* power analysis, larger sample sizes and prospective studies would strengthen the robustness and generalizability of the results. This study primarily focused on short-term outcomes, and future research should include long-term endpoints, such as rebleeding, mortality, and length of hospital stay, to assess the sustained effects of the combination therapy. Although the multicenter design improves external validity, the findings are still limited by patient characteristics and the observational nature of the study. Future studies with more diverse populations are necessary to evaluate the treatment’s applicability across various clinical settings. Additionally, further research should explore optimal dosing regimens, potential side effects, and cost-effectiveness of octreotide combined with pantoprazole to better define its role in clinical practice.

## 5 Conclusion

The combination of octreotide and pantoprazole appears to be associated with a favorable hemostatic effect in elderly patients with peptic ulcers complicated by upper gastrointestinal bleeding. This therapy is associated with a reduction in hemostasis time, improvements in gastric pH and hemoglobin levels, and enhanced coagulation function. These findings suggest that the combined therapy may offer a potential advantage in managing peptic ulcer bleeding in this patient population.

## Data Availability

The raw data supporting the conclusions of this article will be made available by the authors, without undue reservation.
